# What Is Change of Type in Disease?

**Published:** 1858-12

**Authors:** W. O. Markham

**Affiliations:** Physician to St. Mary’s Hospital


"*What is Change of Type in Disease?*" By W. O. MARKHAM, F. R.C.P.L., Physician to St. Mary's Hospital.

What is the meaning of the term "Change of Type in Disease?" It has struck me, in thinking over the answer that should be given to this question, that different persons attach a very different sense to it, and that many who make use of it, not unfrequently do so without holding any very distinct or definite idea of the term. It would, therefore, be well, perhaps, to dwell for a moment on this point, and to endeavor to discover what is the real and proper value which this phrase, "change of type in disease," ought to bring with it. Such a discovery might, I believe, preserve disputants from a great deal of erroneous and superfluous reasonings. I am the more inclined to refer to this subject, because, as far as I know, those who use these now familiar words have not themselves explained the exact meaning which they would infer from them.

The first particular which strikes one on considering this matter is, that the hypothetical change may affix to different objects, viz: to the disease itself, or to the person who is the recipient of the disease, or to both the one and the other at the same time. Now, it cannot be doubted for a moment that some writers who use the phrase, mean thereby to convey to their readers the idea, that an actual change has taken place in the nature of the disease itself—in its intensity, for instance, or some other of its attributes; that there is some particular quality in the morbific element which causes it, when it falls upon or enters into the body, to manifest its presence in a different manner at one period from what it does at another. That such was the idea which Sydenham attached to the epidemics he describes, seems tolerably clear. He found that certain of these diseases, even during the course of a single year, so varied in their characters, that a remedy which, as he says, would cure a man in February, would probably kill him in the following October. It is quite clear that he never meant to infer from this, that the constitution—the bodily condition—of the man had undergone, during the interval between those two months, such a change as would account for the difference in the symptoms of the epidemic at those different periods.

And surely when we now-a-days speak of epidemics and their varieties, we hold very much the same notions as Sydenham did about them, in this respect; and we do so, because we find that, during certain years and at certain periods of the year, epidemics act with greater force and violence, and that their influence reaches more extensively through the people, than they do during other years and at other seasons. An epidemic typhoid fever, or scarlatina, for example, may be at one time of the year what is called "mild" in its character, and the patients attacked by them pass gently and quietly through the exacerbation of the fever, and arrive safely at their cure; at another period, and not a distant one, the same fevers may assume what we call a "malignant" form, and then they fall with peculiar violence on their victims, and with very fatal effects; those who recover from them have often a hard struggle for life, and many succumb during the struggle. Now, it is out of all question to suppose that any changes which the human constitution can have undergone during the short period of time which has elapsed between the visitations of these so different forms of epidemics, can explain the difference in their forms. We trace the change, and in the present state of our knowledge, we are logically compelled to do so, to some peculiarity in the nature of the epidemic, to its intensity or malignancy, as we say metaphorically. These diseases, then, *change their type* quite independently of any change in the constitution of the individual attacked by them; but of course, they may be likewise influenced by any such change in the constitution.

But it is very certain, again, on the other hand, that writers, when they speak of change of type in disease, and especially in reference to the dispute about bleeding in inflammations, very generally mean to infer thereby, the fact of a change in the human constitution itself: they, as I understand them, mean simply this, that the human body is not now constituted exactly as it once was at some previous period, and that it therefore resents diseases, and the remedies applied for their cure, differently from what it did at that previous period. They do not argue, for instance, that pneumonia is in itself a different disease from what pneumonia was fifty years ago; all they maintain is, that the body in which that inflammation manifests itself has undergone some great change. And the proof of the change is founded on the fact, that whereas, in former days, those who suffered under this inflammatiou bore bleeding well, but that now men no longer do so.

Thus, then, it appears that there are, as I have started with saying, two very different ideas attached to this term "change of type;" one idea, supposing that the disease itself is altered in some of its qualities; and the other, that the constitution of the individual in whom it is manifested has undergone a change. And, consequently, any arguments in favor of the theory of a change in type in inflammations, which is derived from a consideration of the different methods of treatment applied at different times to epidemic fevers, cannot have the same value and the same meaning, as those which are founded upon the changes in treatment which pneumonia has undergone in recent times. But authors, and in particular Dr. Christian, argue from the comparison of the effects of treatment of epidemics in past and in present times, that diseases have changed their type, and applies the conclusion to the inflammations of the past and the present times. Epidemic fevers vary in their very nature—this is admitted; but no such distinction has ever been taken in the case of pneumonia or other inflammations. What is asserted in the term "change of type" as applied to pneumonia, is, that the constitution of those now subjected to it differs from what it formerly did. It has never been said, that pneumonia, like fevers, possesses in itself at different times a different character. There is clearly then a very marked distinction between the two cases, and this has not been observed by writers on this subject. Inasmuch, therefore, as epidemic fevers admittedly differ essentially in their nature at different times, having at one time more, and at another less, of an asthenic character, it necessarily follows, that great suspicion must always attach to any conclusions concerning alterations in the constitution of the human body which are derived from a consideration of these fevers, as observed at different and distant epochs. And even in the case of inflammations, is it not also certain, that they also at different times differ in their essential nature, though not in so distinct and well marked a manner as epidemic fevers? We often observe, that pleurisy and pneumonia of one time of the year, or of one year, are much more severe in their character, more asthenic than those of another year, or of another period of it; and surely, to explain this fact, we invoke no change in the human constitution. We do rather, as Sydenham did, seek the change in something *without* the body, in the variable constitution of the epidemic influence. These facts prove the necessity for our having some clearly understood definition of the term "change of type;" and also shows the great difficulty of our drawing any just conclusions from the comparison of the results of treatment of disease at different periods.—*London Medical Times and Gazette.*

